# Quartz Crystal Microbalance Based Sensor Arrays for Detection and Discrimination of VOCs Using Phosphonium Ionic Liquid Composites

**DOI:** 10.3390/s20030615

**Published:** 2020-01-22

**Authors:** Stephanie R. Vaughan, Rocío L. Pérez, Pratap Chhotaray, Isiah M. Warner

**Affiliations:** 1 Department of Chemistry, Louisiana State University, Baton Rouge, LA 70803, USA; svaugh8@lsu.edu (S.R.V.); rperez@lsu.edu (R.L.P.); 2 Department of Chemistry, Indian Institute of Technology Delhi, Delhi 110016, India; pratap.chhotaray2@gmail.com

**Keywords:** VOCs, QCM, ILs, sensor array, MSA, VSA

## Abstract

Herein, we examine two sensing schemes for detection and discrimination of chlorinated volatile organic compounds (VOCs). In this work, phosphonium ionic liquids (ILs) were synthesized and vapor sensing properties examined and compared to phosphonium IL-polymer composites. Pure IL sensors were used to develop a QCM-based multisensory array (MSA), while IL-polymer composites were used to develop an MSA and virtual sensor arrays (VSAs). It was found that by employing the composite MSA, five chlorinated VOCs were accurately discriminated at 95.56%, which was an increase in accuracy as compared to pure ILs MSA (84.45%). Data acquired with two out of three VSAs allowed discrimination of chlorinated VOCs with 100% accuracy. These studies have provided greater insight into the benefits of incorporating polymers in coating materials for enhanced discrimination accuracies of QCM-based sensor arrays. To the best of our knowledge, this is the first report of a QCM-based VSA for discrimination of closely related chlorinated VOCs.

## 1. Introduction

Many volatile organic compounds (VOCs) cause detrimental health and environmental effects after both acute and chronic exposure, which has led to an increase in development of new techniques for detection of these compounds [1,2,3]. However, it is still a challenge to detect and discriminate closely related VOCs. In this regard, electronic noses (e-noses), which mimic the human nose, are of great interest due to a large selection of possible transducers [4,5]. Among such transducers, the quartz crystal microbalance (QCM) coupled with ionic liquids (ILs) has proven to be a viable e-nose [6,7,8,9,10]. The QCM is a sensitive and rapid responding transducer with a large selection of sensing materials, which makes it ideal for fabricating sensor arrays. In this regard, ILs have proven to be good sensing materials due to their tunable properties and ability to be used for detection of a wide range of VOCs [11,12,13]. Briefly, ILs are a class of organic salts with melting points below 100 °C, and by a simple counterion exchange, many properties including toxicity, hydrophobicity, thermal properties, etc. can be tuned [14]. Due to these redeeming qualities, IL-based QCM sensor arrays have proved to be beneficial in vapor sensing studies [15,16,17].

E-noses, or cross-reactive sensor arrays (CRSAs), have two major sensing schemes. The most common is the multisensor array (MSA), which consists of several sensors that are based on chemical affinity. In this scheme, differences in each sensing material allows for interaction with a large range of different VOCs. Each sensor will generate analyte specific response patterns, which can be analyzed using statistical analyses techniques, such as artificial neural networks (ANN), principal component analysis (PCA), cluster analysis (CA), discriminant analysis (DA), etc., in order to identify or discriminate the analyte in question. The second CRSA scheme is the virtual sensor array (VSA), which is based on a single sensor. The VSA generates multiple analyte specific response patterns and can be analyzed in the same manner as an MSA. Fundamentally, the VSA represents a large number of sensors; however, there is only one physical sensor and the remaining “sensors” are imaginary. A schematic of a QCM-based VSA is depicted in Figure S1. In this regard, the VSA reduces cost and complexity of sensing materials as compared to the MSA.

QCM based VSAs were first introduced by Warner, et al. in 2015, and are based on film thickness, viscoelasticity, and harmonics [18]. Briefly, a viscoelastic material is used as the coating material, which results in significantly different behavioral changes under resonant conditions as compared to rigid films due to elastic and viscous properties of the material. This theory is based on the Sauerbrey equation:

$$\Delta f=-\frac{n}{c}\Delta m=-\frac{n}{c}\rho_{f}t_{f}$$

where Δf is change in resonance frequency, n is harmonic number, c is mass sensitivity designated as 17.7 ng cm^−2^ Hz^−1^ for the 5 MHz AT-cut crystal used in this study; $\rho_{f}$ is film density, and $t_{f}$ is film thickness [19]. Thus, the harmonic, thickness, and viscoelasticity of each film will have an effect on sensor response. Harmonics are generated using fundamental frequencies at odd multiples. The quartz crystal resonators (QCRs) used in this work are capable of seven harmonics. In this regard, each harmonic response is recorded and employed as a sensor. For QCM based MSAs and VSAs, the selectivity and sensitivity depend on the coating material.

Herein, a comparative study of QCM based MSAs and VSAs for detection and discrimination of commonly used chlorinated VOCs is described. To accomplish this, three phosphonium-based ILs were synthesized using trihexyltetradecylphosphonium as the cation with three different anions as coating materials for VOC detection. Phosphonium ILs are known to have good chemical stability, viscosity, and the IL trihexyltetradecylphosphonium, in particular, exhibits partial selectivity to a wide range of VOCs [15,20,21]. Composite materials were then created using an IL-polymer blend with the phosphonium ILs and polydimethylsiloxane (PDMS). PDMS is known to increase sensitivity of gas sensors [22], and IL-polymer blends have been shown to increase discrimination of VOCs due to enhanced viscoelastic properties [23,24]. In order to investigate the vapor sensing properties of each IL and IL-PDMS composite, thin films of each were deposited on the surface of QCRs via electrospray deposition and subsequently exposed to a set of five chlorinated VOCs. Each set of sensors (pure IL and composites) exhibited cross reactive patterns and were determined to be suitable for MSA fabrication. The resulting data from each set of sensors (pure IL sensors and composite sensors) were then used to develop statistical models for discrimination of five VOCs. PCA was used to assess the dimensionality of each data set and to obtain a visual representation of separation among the chlorinated VOCs. DA was used to develop predictive models for discriminating chlorinated compounds. Lastly, each composite sensor exhibited multiple harmonic responses and each data set was used to fabricate three different VSAs.

## 2. Materials and Methods

### 2.1. Materials

Trihexyltetradecylphosphonium (P_66614_) chloride, sodium dodecylbenzenesulfonate (DBS), chloropropane, chlorobutane, and tetrachloromethane were purchased from Sigma-Aldrich (St. Louis, MO, USA). Sodium benzenesulfonate (BS) and polydimethylsiloxane (PDMS) were purchased from Acros Organics (West Chester, PA, USA). Sodium 4-n-octylbenzenesulfonate (OBS) was purchased from Alfa Aesar (Haverhill, MA, USA), dichloromethane (DCM) was purchased from BDH VWR Analytical (Radnor, PA, USA), and chloroform was purchased from Macron Fine Chemicals (Center Valley, PA, USA). All chemicals were used as purchased without further purification.

### 2.2. Instrumentation

A Q-Sense QCM-D E4 system and associated QCRs were used for these studies and previously purchased from Biolin Scientific (Stockholm, Sweden). Each QCR is an AT-cut gold-coated quartz crystal with a diameter of 14 mm, thickness of 0.3 mm and fundamental frequency of 4.95 MHz ± 50 kHz. Both readout equipment (Model 5878) and mass flow controllers (Model 5850E) were obtained from Brooks Instrument, LLC (Hatfield, PA, USA).

### 2.3. Synthesis and Characterization of ILs

Three ILs were synthesized using a biphasic ion exchange reaction. As an example of a typical synthetic procedure, [Na][DBS] was dissolved in water, while [P_66614_][Cl] was dissolved in DCM at a 1:1 mole ratio. Prepared solutions were mixed together and allowed to stir for 48 h to obtain [P_66614_][DBS]. After completion of ion exchange, NaCl (byproduct) was removed from the DCM layer by washing with water several times. To isolate the final product, DCM was removed using rotary evaporation followed by lyophilization to remove any residual water. The reaction procedure referenced above was used to obtain remaining ILs by reacting [P_66614_][Cl] with [Na][BS], and [Na][OBS] to obtain [P_66614_][BS], and [P_66614_][OBS], respectively. All three ILs were colorless and viscous liquids. Structures of starting materials are shown in Figure S2.

Electrospray ionization mass spectrometry (ESI-MS) and Fourier transform infrared spectrometry (FT-IR) were used to characterize ILs. ESI-MS was accomplished using an Agilent 6210 system in positive and negative ion modes. FT-IR was performed using a Bruker Alpha & Tensor 27 FT-IR instrument.

### 2.4. Preparation of IL Stock Solutions

Stock solutions of [P_66614_][DBS], [P_66614_][BS], and [P_66614_][OBS] (1 mg/mL) were prepared using DCM in 20 mL borosilicate glass scintillation vials.

### 2.5. Preparation of Composite Stock Solutions

Stock solutions of [P_66614_][DBS] (1 mg/mL) with PDMS (0.5 mg/mL), [P_66614_][BS] (1 mg/mL) with PDMS (0.5 mg/mL), and [P_66614_][OBS] (1 mg/mL) with PDMS (0.5 mg/mL) were prepared using DCM in 20 mL borosilicate glass scintillation vials.

### 2.6. Preparation of Sensing Films

Prior to coating, each QCR was cleaned using RCA standard clean 1 solution (5:1:1 deionized water, 30% hydrogen peroxide, and ammonium hydroxide) [25]. An electrospray method was used for deposition of ILs and composites onto each QCR surface. Parameters for electrospray remained constant for each thin film: flowrate of 100 µL/min, current of 30 µA, voltage of 16.6 kV and a working distance of 7 cm. After coating, films were dried with nitrogen and then stored in a desiccator prior to use. The change in frequency between coated and uncoated QCRs in all of the studied ILs and composites was maintained at ~ −2000 Hz. Once coated with materials, QCRs are referred to as sensors.

### 2.7. Data Collection

Each analyte was introduced at five different instrumentally controlled dilutions of flow rate ratios (0.05, 0.1, 0.2, 0.3, and 0.4 *F_s_*/*F_tot_*) that correspond to 5%, 10%, 20%, 30%, and 40% of saturated vapor pressure in a 20 mL vial of VOC and argon gas. To achieve this, a flow system that consisted of two independent gas flow channels, one for analyte vapors and another for carrier gas, was used. Prior to data collection, the system was purged with ultrapure argon to achieve a stable baseline. Subsequently, a vial containing the VOC of choice was bubbled with argon to generate a sample of equilibrated headspace. The analyte and carrier channels merged to allow dilution of the analyte flow to yield respective flow rate ratios [26]. The total flow rate was held constant at 100 sccm by using digital mass flow controllers. VOC vapors mixed across 1-m length of tubing and then flowed over each sensor. To remove analyte vapors, the system was purged with argon at room temperature until the baseline was recovered. A schematic of the system described has been previously published and is provided in Figure S3 [26].

### 2.8. Data Analysis

Multiple harmonic data were generated from vapor sensing studies expressed by change in frequency (Δf) in units of hertz (Hz). PCA was used to assess the dimensionality of the observed sensor data (MSA and VSAs) and to obtain a visual representation of separation among the chlorinated compounds with respect to the principal components. DA was used to develop a predictive model for distinguishing chlorinated VOCs, using the principal components as predictor variables.

## 3. Results

### 3.1. Characterization of ILs

Each IL was confirmed using ESI-MS (Figures S4–S6) and FT-IR (Figures S7–S9). All three ILs were liquids at room temperature; thus thermal properties were not investigated.

### 3.2. Evaluation of IL Sensor Responses

**Figure 1:**
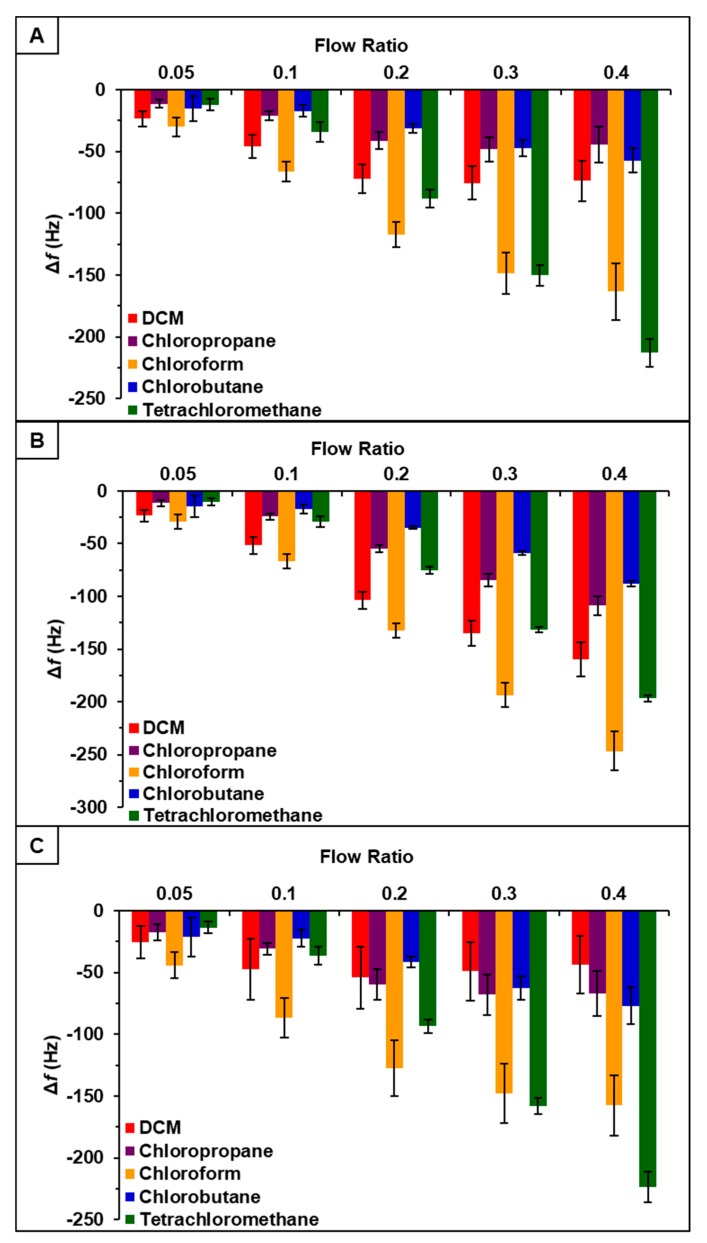
Sensor response of chlorinated VOCs at five flow ratios for (**A**) [P_66614_][DBS], (**B**) [P_66614_][BS], and (**C**) [P_66614_][OBS]. Error bars represent standard deviation for three replicate measurements.

Vapor sensing properties of [P_66614_][DBS], [P_66614_][BS], and [P_66614_][OBS] were evaluated by inserting three QCM sensors coated with respective ILs into QCM-D chambers. Collectively sensors were exposed to a set of five chlorinated VOCs, which included dichloromethane, chloroform, chloropropane, chlorobutane, and tetrachloromethane, at five different instrumentally controlled sample flow rate ratios (0.05, 0.1, 0.2, 0.3, and 0.4 *F_s_*/*F_tot_*). Changes in resonance frequency were measured by exposing sensors to individual VOCs at indicated flow rate ratios for 3-min intervals for a total exposure time of approximately 15 min. Three replicate measurements were completed for each VOC. Sensor responses for [P_66614_][DBS], [P_66614_][BS], and [P_66614_][OBS] are presented in Figure 1 expressed as change in frequency (Δ*f*) versus flow rate ratios. While each sensor exhibited reversible sorption and a stable starting baseline, some sensor drift occurred over the course of the experiment. Furthermore, all sensors exhibited reproducible responses with the exception of low flow ratios (0.05 and 0.1), which resulted in large standard deviations (Figure 1). It should also be noted that [P_66614_][OBS] exhibited poor reproducibility in response to dichloromethane across all flow ratios. Based on pattern responses observed in Figure 1, fabrication of a MSA is possible, and these results are discussed in Section 4.1. In an attempt to increase sensor response and reproducibility at low flow ratios, incorporation of PDMS with ILs to create composite materials was investigated.

### 3.3. Evaluation of Sensor Responses for Composites

**Figure 2:**
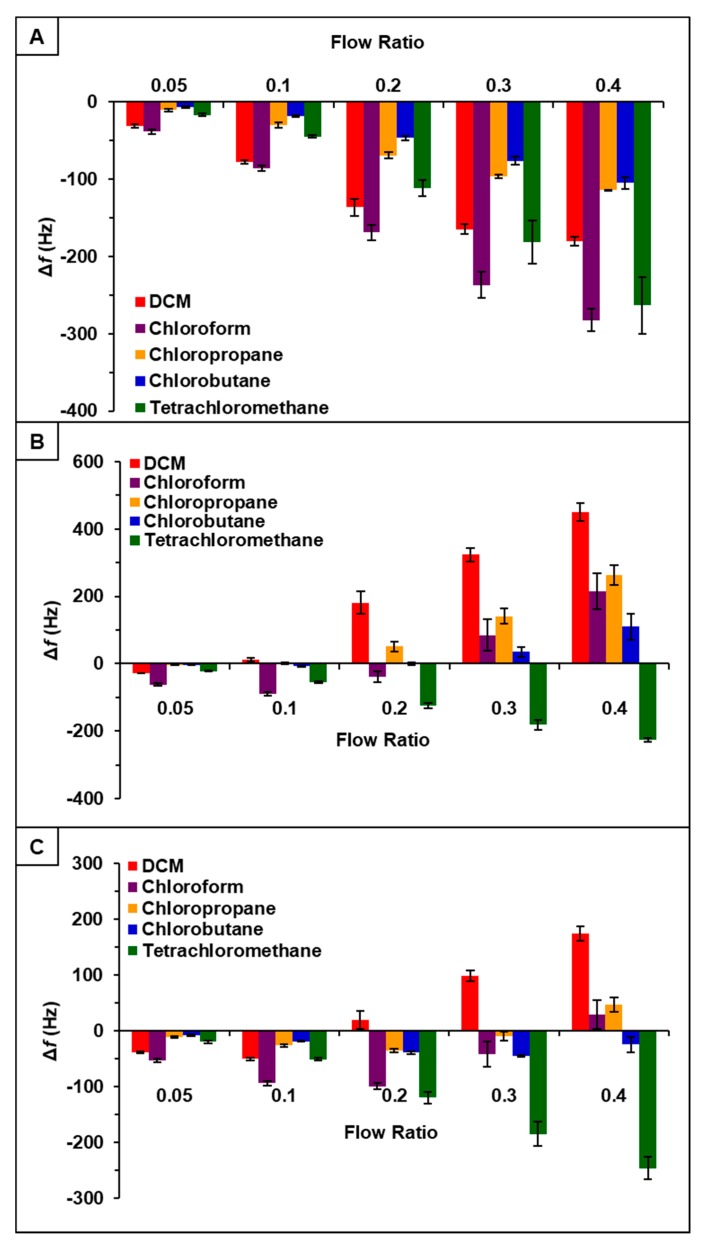
Sensor response of chlorinated VOCs at five flow ratios for (**A**) [P_66614_][DBS]-PDMS, (**B**) [P_66614_][BS]-PDMS, and (**C**) [P_66614_][OBS]-PDMS. Error bars represent standard deviation for three replicate measurements.

It was hypothesized that incorporation of PDMS with phosphonium ILs would increase sensor response to chlorinated compounds [22]. Thus, the vapor sensing properties of [P_66614_][DBS]-PDMS, [P_66614_][BS]-PDMS, and [P_66614_][OBS]-PDMS were evaluated using similar parameters as ILs studies. Briefly, three QCM sensors coated with respective IL-polymer composites were inserted into QCM-D chambers and exposed to the same set of chlorinated VOCs at identical flow ratios. Similar to IL studies, sensors were exposed to VOCs at indicated flow ratios for 3 min intervals for a total exposure time of approximately 15 min with three replicate measurements. Sensor responses for [P_66614_][DBS]-PDMS, [P_66614_][BS]-PDMS, and [P_66614_][OBS]-PDMS are presented in Figure 2 expressed as change in frequency (Δf) versus flow rate ratios. All sensors were found to be reusable, which is consistent with each sensor exhibiting a stable baseline and reversible sorption, as shown in Figure S10. Moreover, each sensor produced analyte specific response patterns as compared to each other, as well as to their IL counterparts.

With respect to [P_66614_][DBS], [P_66614_][DBS]-PDMS exhibited similar response patterns; however, there was an increase in overall sensor response, as well as smaller error bars with all analytes except tetrachloromethane. Overall, response patterns generated from IL-PDMS composites showed enhanced reproducibility and increased sensor response to chlorinated compounds, with the exception of tetrachloromethane.

**Figure 3:**
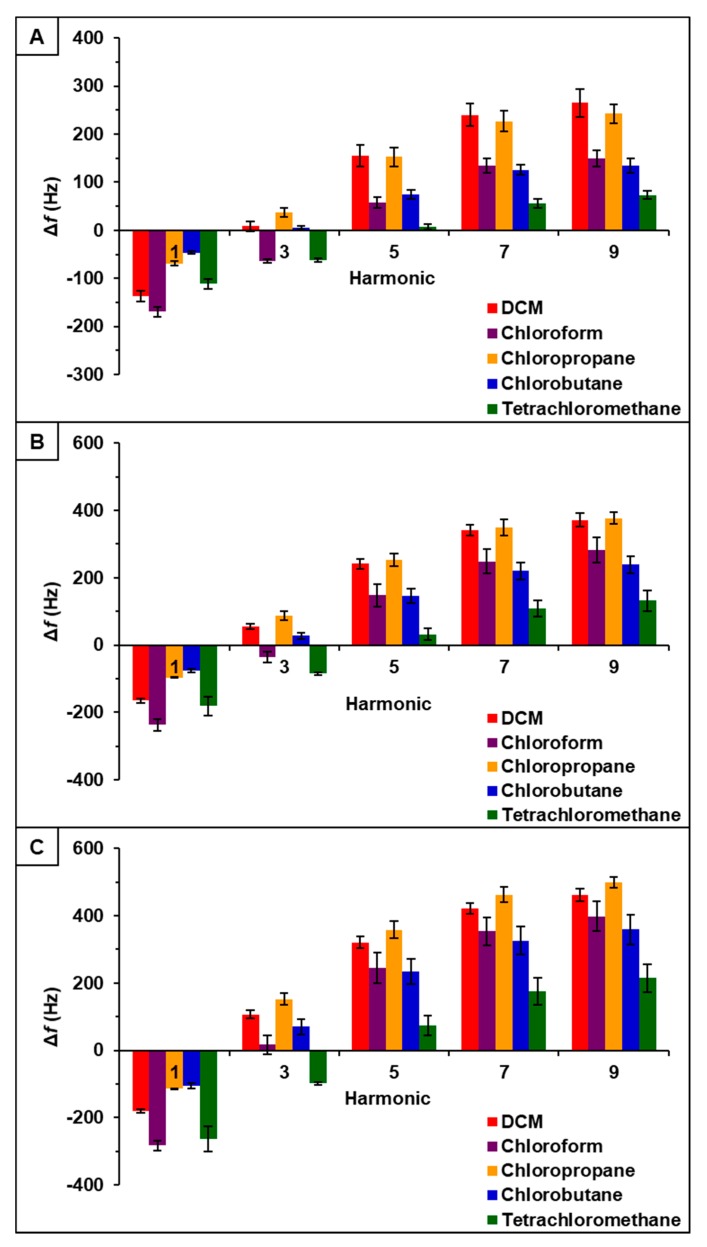
[P_66614_][DBS]-PDMS sensor response to chlorinated VOCs at multiple harmonics at (**A**) 0.2 flow ratio, (**B**) 0.3 flow ratio, and (**C**) 0.4 flow ratio. Error bars represent standard deviation for three replicate measurements.

**Figure 4:**
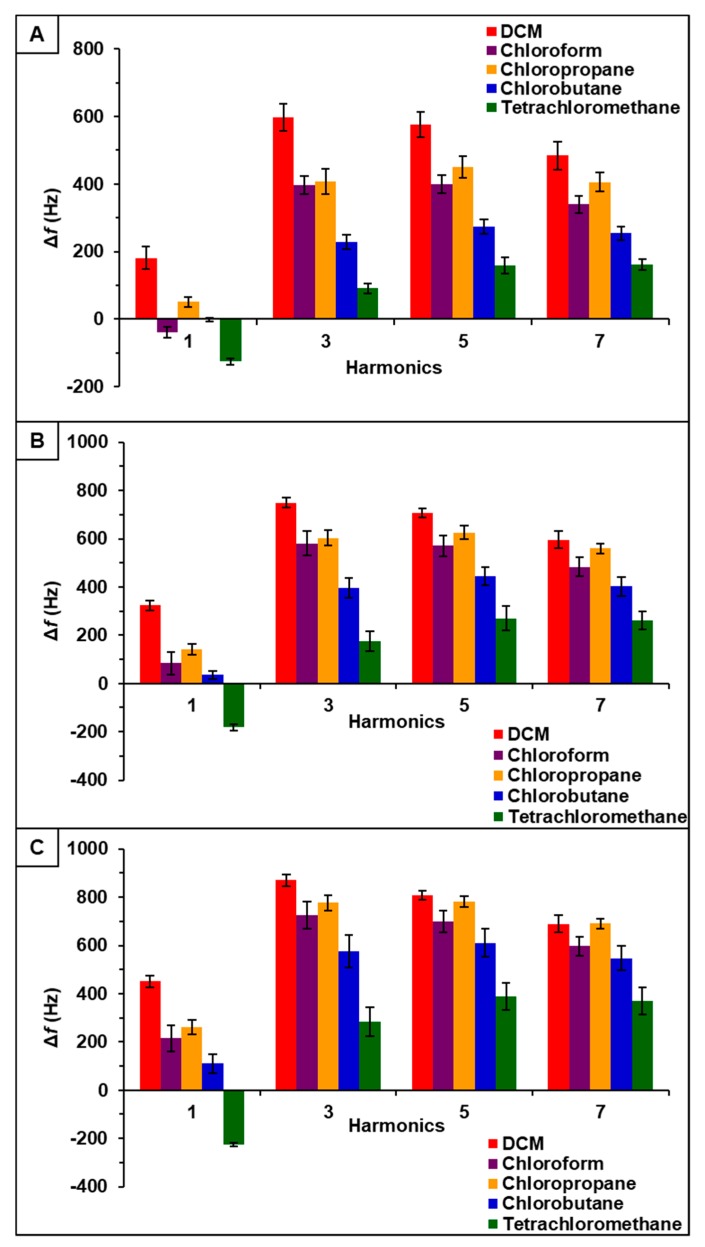
[P_66614_][BS]-PDMS sensor response to chlorinated VOCs at multiple harmonics at (**A**) 0.2 flow ratio, (**B**) 0.3 flow ratio, and (**C**) 0.4 flow ratio. Error bars represent standard deviation for three replicate measurements.

**Figure 5:**
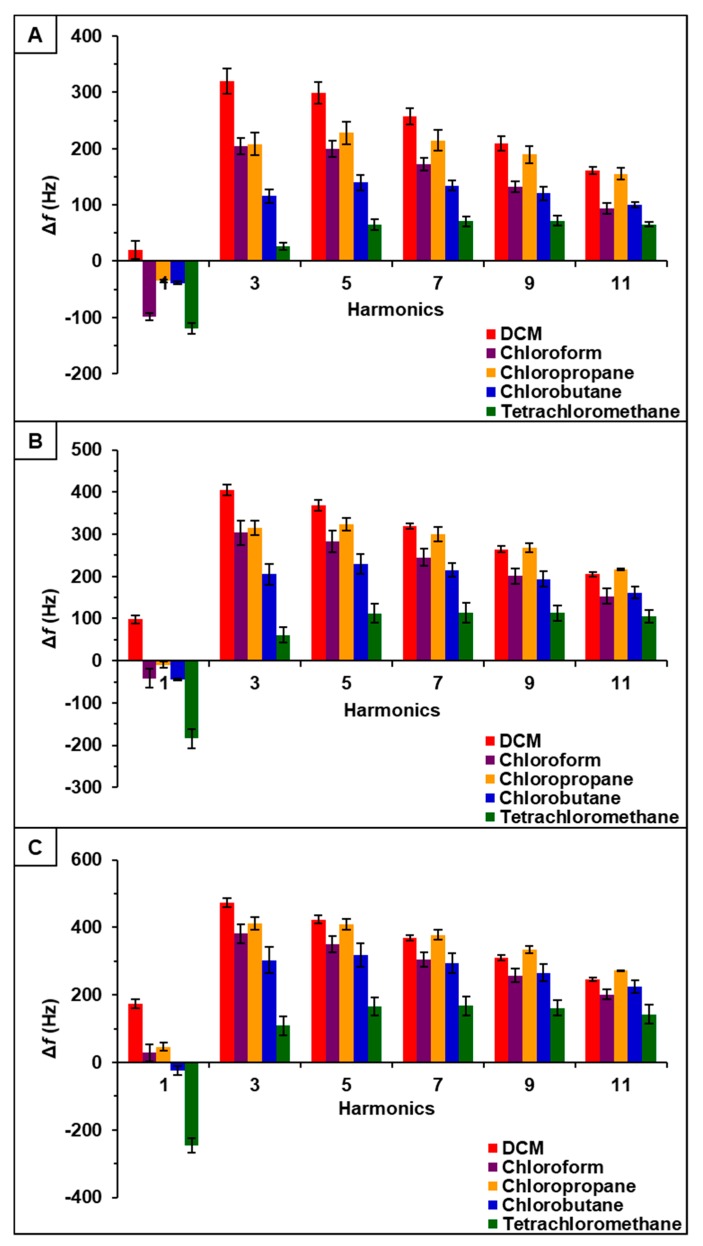
[P_66614_][OBS]-PDMS sensor response to chlorinated VOCs at multiple harmonics at (**A**) 0.2 flow ratio, (**B**) 0.3 flow ratio, and (**C**) 0.4 flow ratio. Error bars represent standard deviation for three replicate measurements.

Observation of data from [P_66614_][BS]-PDMS showed an entirely different response pattern as compared to [P_66614_][BS]. [P_66614_][BS]-PDMS exhibited both positive and negative changes in frequency, whereas all responses were negative values for [P_66614_][BS]. Interestingly, sensor responses for chloropropane and chlorobutane were negligible at lower flow ratios, whereas pure IL sensor generated significantly larger responses. Notably, tetrachloromethane was the only compound to achieve a negative changes in frequency over all five flow ratios. Similar to [P_66614_][BS]-PDMS, [P_66614_][OBS]-PDMS exhibited positive and negative changes in frequency and tetrachloromethane achieved negative values over all flow ratios. In contrast to [P_66614_][BS]-PDMS, [P_66614_][OBS]-PDMS exhibited an overall lower sensor response. It is noted that composite sensors exhibited multiple harmonic responses, which was not exhibited by pure IL sensors. Figure 3, Figure 4 and Figure 5 depict sensor responses across multiple harmonics for [P_66614_][DBS]-PDMS, [P_66614_][BS]-PDMS, and [P_66614_][OBS]-PDMS respectively. The positive and negative shifts in resonant frequency can be attributed to incorporation of PDMS, which changes the viscoelasticity of the sensor coating [27]. Based on pattern responses observed in Figure 3, Figure 4 and Figure 5 fabrication of a MSA and VSA are possible and these results will be discussed in Section 4.1 and Section 4.2.

## 4. Discussion

### 4.1. Evaluation of MSAs

Based on pattern responses observed in Figure 1 and Figure 2, fabrication of two MSAs to discriminate between the chlorinated compounds was possible. The first MSA was developed using sensor responses from pure IL sensors, [P_66614_][DBS], [P_66614_][BS], and [P_66614_][OBS]. The second array was developed using sensor responses from composite sensors, [P_66614_][DBS]-PDMS, [P_66614_][BS]-PDMS, and [P_66614_][OBS]-PDMS. To achieve the first array, the raw Δf data collected from the pure IL sensors of the first harmonic were used to develop a predictive model using DA. The hypothesis that the covariance matrices associated with the three sensor variables were the same across all VOCs was strongly rejected (*p*-value < 0.0001). Thus, quadratic DA (QDA) was used, which fits a model that estimates the covariance matrices separately for each VOC [26]. The composite MSA was achieved using the same parameters.

**Figure 6:**
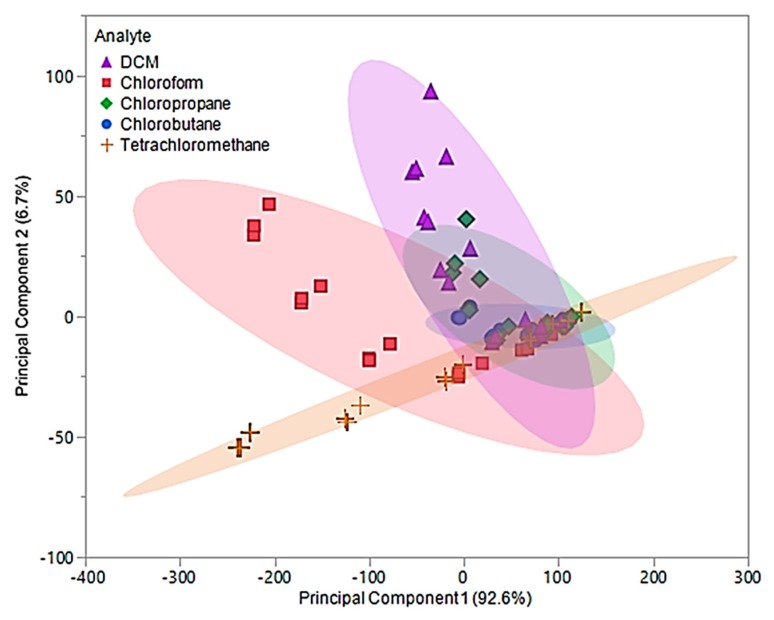
Principal component plot for discrimination of five chlorinated VOCs with respect to a three sensor MSA. The plot considers 75 total measurements consisting of three replicate measurements at five different flow ratios for each VOC (15 measurements per sample) using pure IL sensors.

For pure IL MSA, the first two principal components accounted for 99.3% of the variability in the three predictors. The first principal component, which accounted for 92.6% of the variability, represents the sum of the three sensor responses. While the second principal component represents a comparison between [P_66614_][BS] and [P_66614_][OBS] responses, which accounted for 6.7% of the total variation. Figure 6 depicts a plot of the first two principal component scores, where some visual separation between DCM, chloroform, and tetrachloromethane is provided. However, the first two principal components do not provide any visual separation between chlorobutane and chloropropane, and there is severe overlap between chlorobutane, chloropropane and remaining VOCs.

Based on this plot, it is suggested that there will be difficulty distinguishing between these VOCs, especially between chlorobutane and chloropropane with the model produced by DA. The values for the first two principal components were used as predictor variables in QDA. The QDA predictive model resulted in 30 misclassifications, corresponding to an error rate of 40%. Of these misclassifications, six DCM measurements were misclassified as chlorobutane, two as chloropropane, one chloroform measurement was misclassified as chlorobutane, one as chloropropane, four as tetrachloromethane, nine chloropropane measurements were misclassified as chlorobutane, one as DCM, one chlorobutane was misclassified as chloropropane, and five tetrachloromethane measurements were misclassified as chlorobutane. This corresponded to an overall accuracy of 60%. With an excess of misclassifications and low accuracy, the discriminate scores from the QDA model were further investigated. It was found that majority of these classifications were occurring in the 0.05 and 0.1 flow ratios across all VOCs. Thus, new principal components using Δ*f* measurements from 0.2, 0.3, and 0.4 flow ratios were evaluated and used to develop an optimized QDA model.

**Figure 7:**
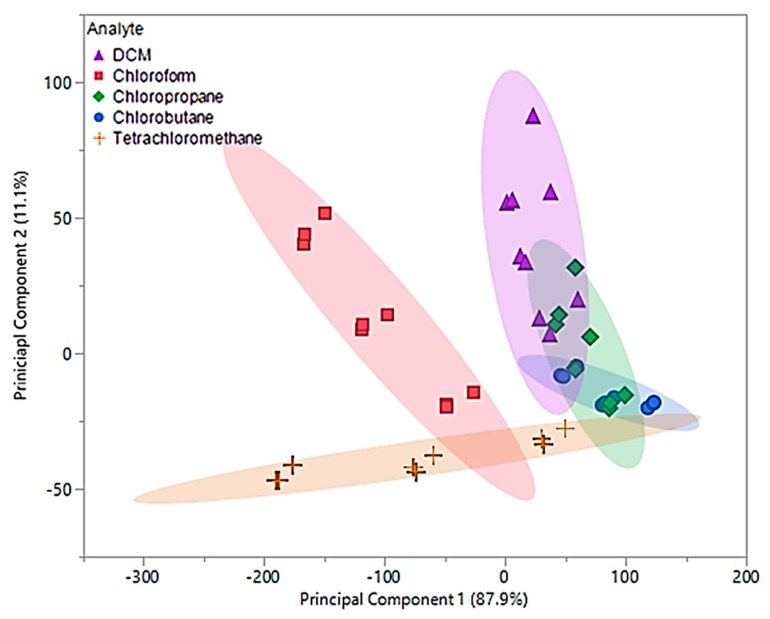
Principal component plot for discrimination of five chlorinated VOCs with respect to a three sensor MSA. Plot considers 45 total measurements consisting of three replicate measurements at three different flow ratios for each VOC (nine measurements per sample) using pure IL sensors.

Data obtained from 0.2–0.4 flow ratios demonstrated that the first two principal components accounted for 99% of the total variability in the three predictors. The first principal component accounted for 87.9% of the variability and similar to the original principal components, represents the sum of the three sensor measurements. Similar to the original principal components, the optimized second component represents a comparison between [P_66614_][BS] and [P_66614_][OBS] measurements, but accounts for 11.1% of the total variability. Based on optimized PCA plot shown in Figure 7, an improvement in visual separation between tetrachloromethane and DCM, tetrachloromethane and chloroform, and between DCM and chloroform is provided. However, the optimized components are still unable to provide visual separation between chloropropane and chlorobutane, and an overlap of chlorobutane, chloropropane, DCM and tetrachloromethane is observed. This optimized PCA plot suggests that there may be difficuly discriminating between these VOCs, but improvement in discrimination as compared to the original PCA plot in Figure 6. To test this theory, optimized principal components were used as predictor variables in QDA. The optimized QDA model resulted in a total of seven misclassifications, which corresponds to an error rate of 15.55%. Misclassifications consisted of one DCM measurement classified as chloropropane, four chloropropane classified as chlorobutane, one chloropropane classified as DCM, and one chlorobutane classified as chloropropane. Overall accuracy of the optimized QDA model was 84.45%, which was a large improvement as compared to the original model. It should be noted that all tetrachloromethane and chloroform measurements were accurately classified as suggested using the PCA plot in Figure 7.

**Figure 8:**
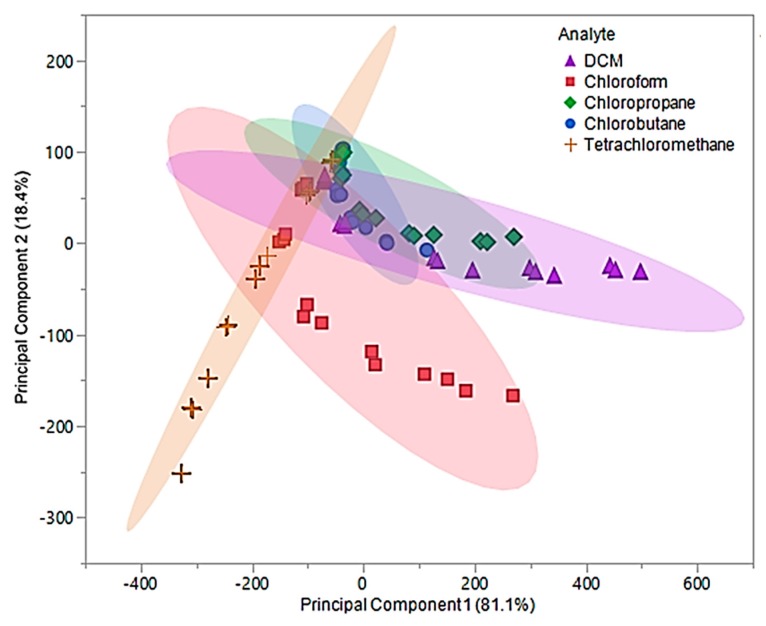
Principal component plot for discrimination of five chlorinated VOCs with respect to a three sensor MSA. Plot considers 75 total measurements consisting of three replicate measurements at five different flow ratios for each VOC (15 measurements per sample) using composite sensors.

Upon examination of the composite MSA, 99.5% of the total variability in the three predictors was accounted for using the first two principal components. The first principal component accounted for 81.1% of variance and represented the sum of the three sensor responses. The second principal component, which accounted for 18.4% of the variability, represented a comparison between [P_66614_][DBS]-PDMS and [P_66614_][BS]-PDMS responses. Based on Figure 8, it was proposed that using the predicative QDA model will result in VOCs being misclassified as chloropropane or chlorobutane.

This hypothesis is the result of significant overlap of chloropropane and chlorobutane with DCM, chloroform, and tetrachloromethane. This proposal was evaluated using the first two principal components as predictor variables in QDA. The QDA model had an error rate of 36%, which accounted for 27 misclassifications. These misclassifications were comprised of five DCM measurements classified as chlorobutane, six chloroform measurements classified as tetrachloromethane, nine chloropropane measurements classified as chlorobutane and three as DCM, one chlorobutane classified as DCM, and three tetrachloromethane measurements classified as chlorobutane. This model was found to have an accuracy of 64%, which lead to further investigation of the discriminate scores. Similar to the original pure IL MSA, most of the misclassifications were due to the lower flow ratios (0.05 and 0.1). Therefore, new principal components using Δf measurements from 0.2, 0.3, and 0.4 flow ratios were evaluated and used to develop an optimized QDA model.

**Figure 9:**
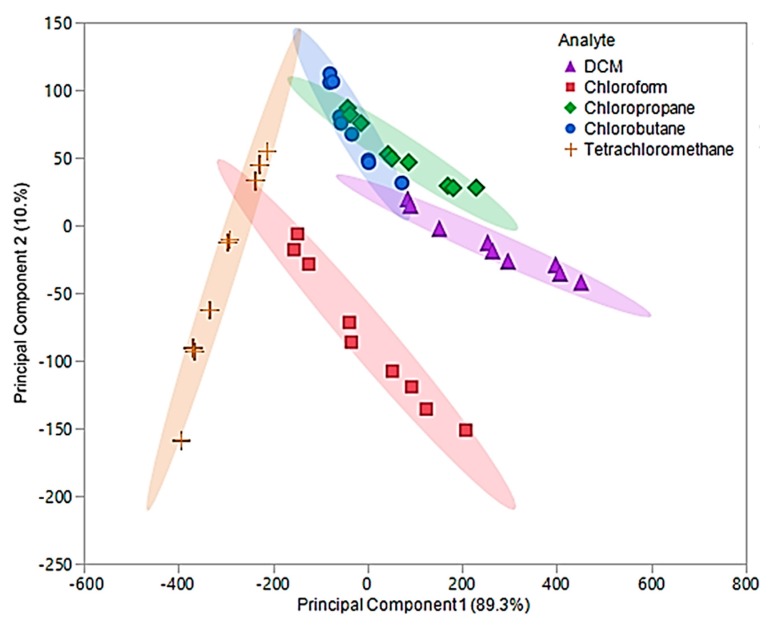
Principal component plot for discrimination of five chlorinated VOCs with respect to a three sensor MSA. The plot considers 45 total measurements consisting of three replicate measurements at three different flow ratios for each VOC (nine measurements per sample) using composite sensors.

In this examination, the first two principal components accounted for 99.6% of the total variability in the three predictors and represented the same factors as the original components. The optimized first principal component accounted for 89.3% of the variability, while the second component accounted for 10.3%. An optimized PCA plot is depicted in Figure 9, where enhanced visual separation between tetrachloromethane, chloroform, and DCM is provided. Nonetheless, poor visual separation persisted between chlorobutane and chloropropane of the optimized principal components.

The optimized principal components were used as predictor variables to develop the optimized QDA model. With the exception of two measurements, this model accurately discriminated between the five chlorinated VOCs and resulted in an error rate of 4.44%. The misclassification was due to two chloropropane measurements being classified as chlorobutane. As previously mentioned, chloropropane and chlorobutane overlapped in the optimized PCA plot (Figure 9). Thus, this misclassification was not alarming. The overall accuracy of this model was determined to be 95.56%, which is a drastic improvement over the original QDA model as well as the pure IL QDA model.

### 4.2. Evaluation of VSAs

[P_66614_][DBS]-PDMS, [P_66614_][BS]-PDMS, and [P_66614_][OBS]-PDMS exhibited sensor responses across multiple harmonics, as shown in Figure 3, Figure 4 and Figure 5 respectively. To evaluate the capability of VSAs for discrimination of chlorinated VOCs, each sensor was analyzed as an independent system. To accomplish this task, raw changes in frequency (Δf) data collected from each sensor across multiple harmonics was used to develop a predictive model using QDA. [P_66614_][DBS]-PDMS exhibited five harmonics (1st, 3rd, 5th, 7th, and 9th), [P_66614_][BS]-PDMS exhibited four harmonics (1st, 3rd, 5th, and 7th), and [P_66614_][OBS]-PDMS exhibited six harmonics (1st, 3rd, 5th, 7th, 9th, and 11th). For each sensor, the hypothesis that the covariance matrices associated with the five, four, and six sensor variables, respectively, were the same across all VOCs was strongly rejected (*p*-value < 0.0001). Thus, QDA was used, which fits a model that estimates the covariance matrices separately for each VOC [26]. Based on optimization of the composite MSA, these QDA models consider only Δf measurements for 0.2, 0.3, and 0.4 flow ratios.

In regards to [P_66614_][DBS]-PDMS, four principal components were used as predictor variables to develop the QDA model. This model resulted in 100% accuracy in discriminating the five chlorinated VOCs. In contrast, [P_66614_][BS]-PDMS, used three principal components as input variables for QDA, which resulted in 91.11% discrimination accuracy. These misclassifications consisted of one chlorobutane measurement being classified as chloropropane, and three chloropropane measurements classified as chlorobutane. Lastly, [P_66614_][OBS]-PDMS used five principal components as predictor variables for development of the QDA model, which resulted in 100% accuracy. Due to these models using more than two principal components, and hence three-dimensional or more, it is not possible to illustrate the score plots. For simplicity, two-dimensional QDA canonical plots for each VSA are provided in Figures S11–S13.

## 5. Conclusions

In this study, two novel phosphonium ILs and one previously reported phosphonium IL were synthesized and their vapor sensing properties were investigated using a QCM based MSA. To further evaluate the vapor sensing properties of these ILs, PDMS was incorporated to create composite materials. The incorporation of PDMS resulted in significantly different sensor responses from pure ILs. Ultimately, composite materials vapor sensing properties were investigated using a QCM based MSA and VSA. It was found that pure ILs and composite materials were not useful for vapor detection of chlorinated VOCs at low flow ratios (0.05 and 0.1). However, by employing the composite MSA, five chlorinated VOCs were accurately discriminated at 95.56%, which was an increase in accuracy as compared to pure ILs MSA (84.45%). It should be noted that pure ILs were not capable of VSA fabrication, while composite sensors were capable. With the exception of [P_66614_][BS]-PDMS (91.11%), VSAs exhibited higher accuracies than the MSA at 100%. Although further studies need to be investigated to fully understand vapor interaction with sensing materials, these studies have provided greater insight into benefits of incorporating polymers for enhanced discrimination accuracies of QCM based sensor arrays. These sensor arrays are currently in the basic research stage; however, they show promise for potential use in laboratories where color additives and inks are produced to monitor chlorinated VOC exposure to employees.

## Supplementary Materials

The following are available online at https://www.mdpi.com/1424-8220/20/3/615/s1, Figure S1. Schematic of QCM-based VSA, Figure S2. Structures of starting materials used to synthesize ILs, Figure S3. Schematic of QCM flow system, Figure S4. Electrospray ionization mass spectrometry (ESI-MS) spectra for [P_66614_][DBS], Figure S5. Electrospray ionization mass spectrometry (ESI-MS) spectra for [P_66614_][BS], Figure S6. Electrospray ionization mass spectrometry (ESI-MS) spectra for [P_66614_][OBS], Figure S7. Fourier transform infrared (FT-IR) spectra for [P_66614_][DBS], Figure S8. Fourier transform infrared (FT-IR) spectra for [P_66614_][BS], Figure S9. Fourier transform infrared (FT-IR) spectra for [P_66614_][OBS], Figure S10. Sensorgram for first replicate measurement, Figure S11. Canonical plot for discrimination of five chlorinated VOCs with respect to a five sensor VSA, Figure S12. Canonical plot for discrimination of five chlorinated VOCs with respect to a four sensor VSA, Figure S13. Canonical plot for discrimination of five chlorinated VOCs with respect to a six sensor VSA.
