# Supplementary material for: Quartz Crystal Microbalance Based Sensor Arrays for Detection and Discrimination of VOCs Using Phosphonium Ionic Liquid Composites
Source: Sensors (Basel). 2020 Jan 22;20(3):615. doi: 10.3390/s20030615 (PMC7037595; doi:10.3390/s20030615)
Supplement: Supplementary file 1 [file sensors-20-00615-s001.pdf]

Supplementary Materials

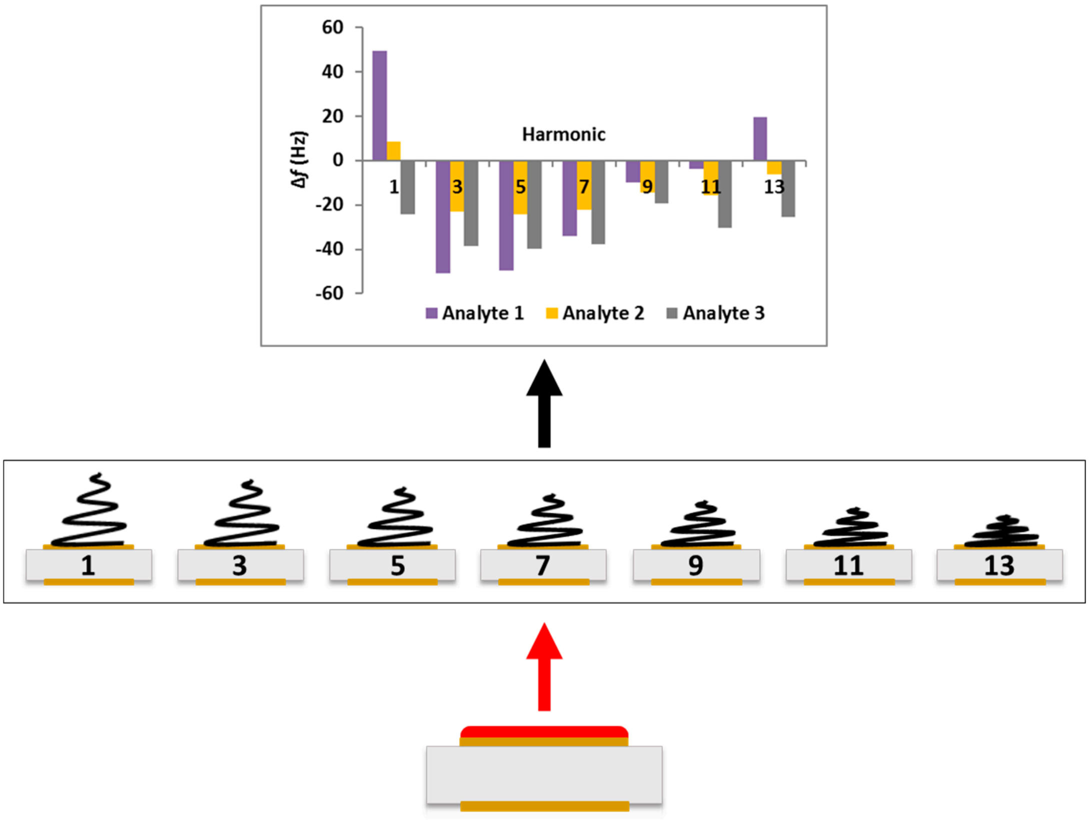

Figure S1. Schematic of QCM-based VSA. Red represents one chemically coated sensor that produces multiple responses. Smaller sensors labeled 1 – 13 represent "imaginary" sensors. The graph is a visual representation of the analyte specific responses generated by each sensor.

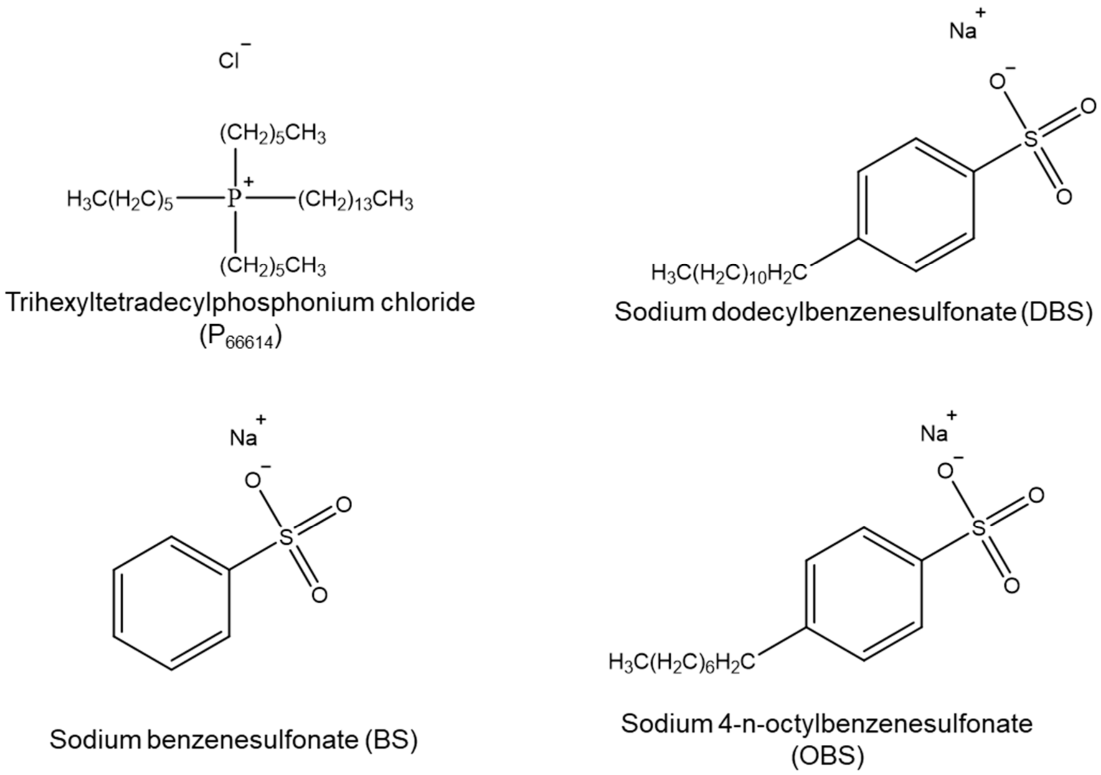

Figure S2. Structures of starting materials used to synthesize ILs

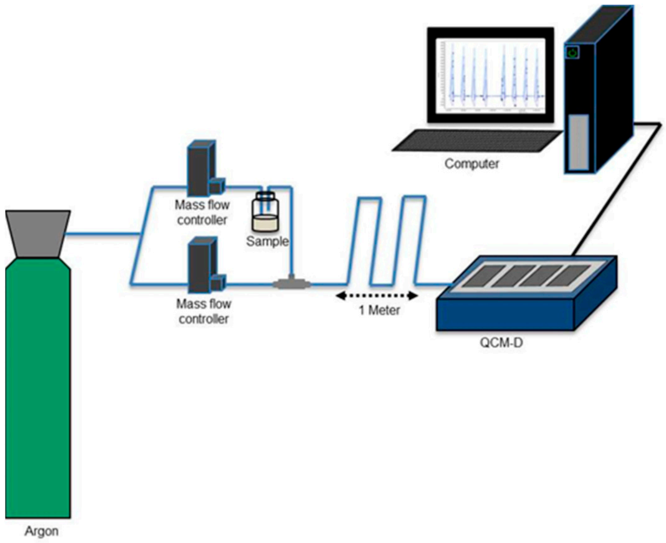

Figure S3. Schematic of QCM flow system.

.

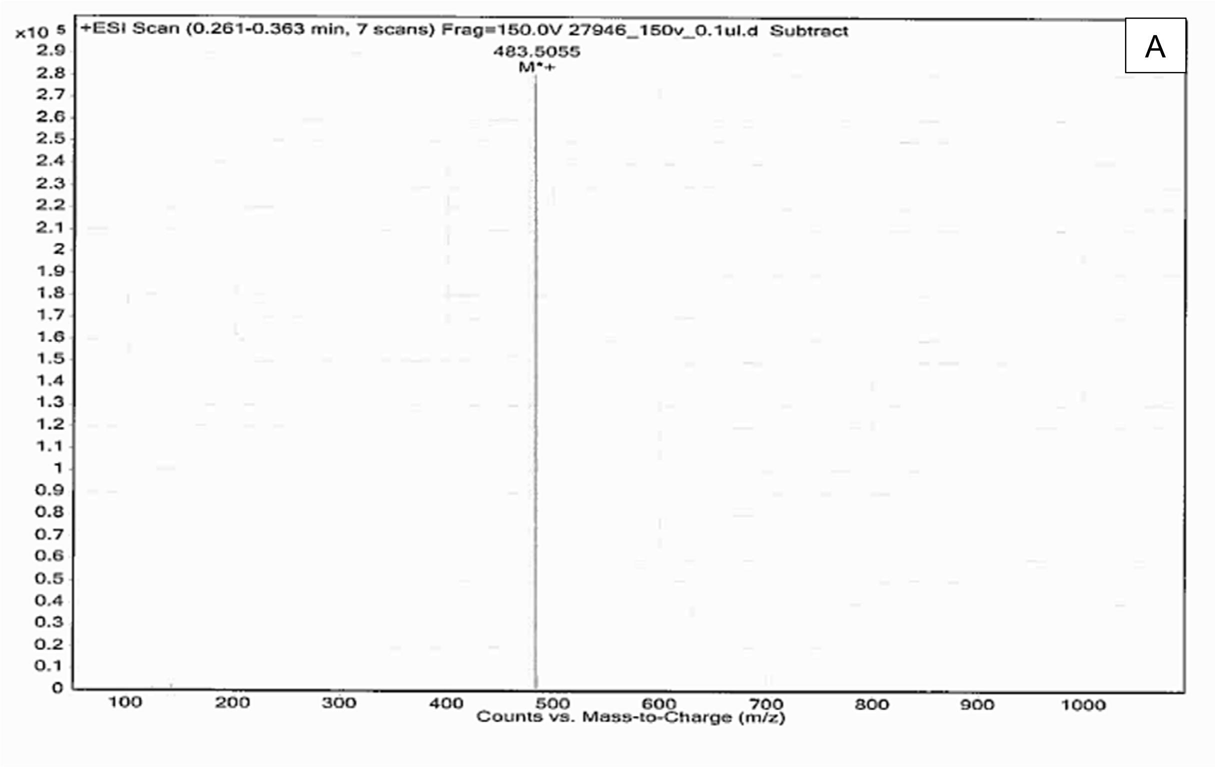

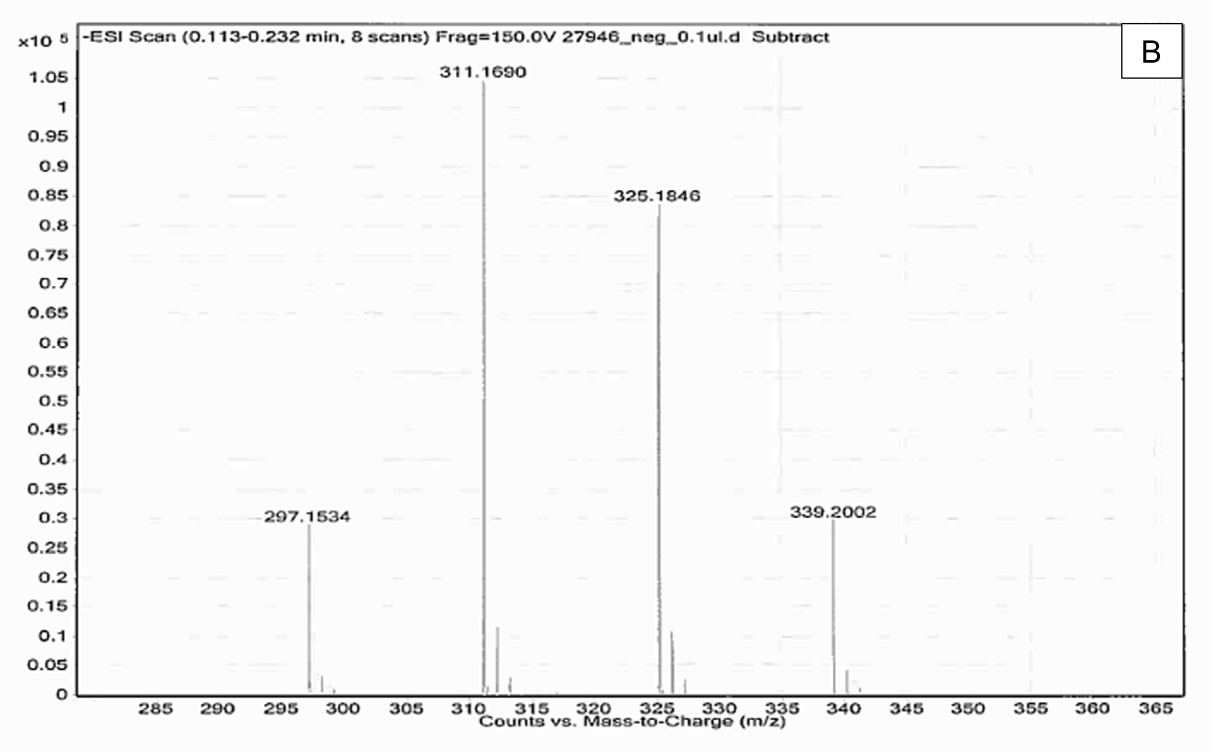

Figure S4. Electrospray ionization mass spectrometry (ESI-MS) spectra for [P_{66614}][DBS], where A) is in positive mode and B) is in negative mode. Solvent used was DCM.

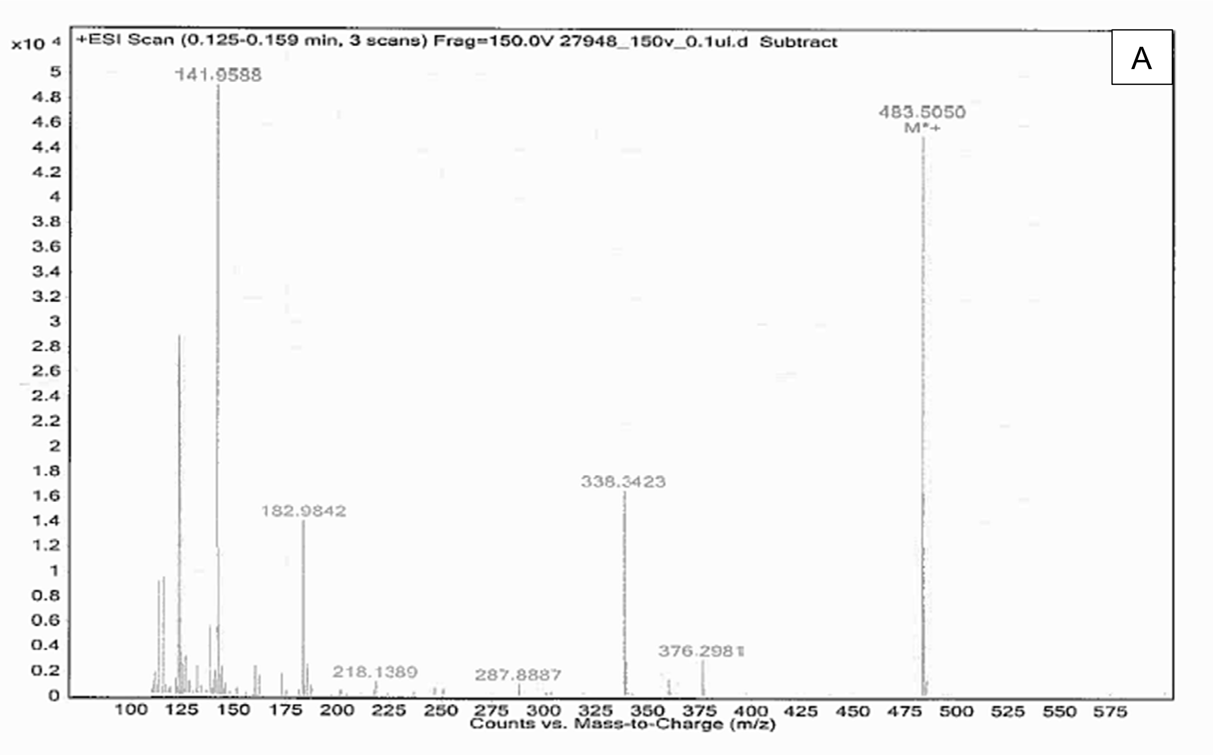

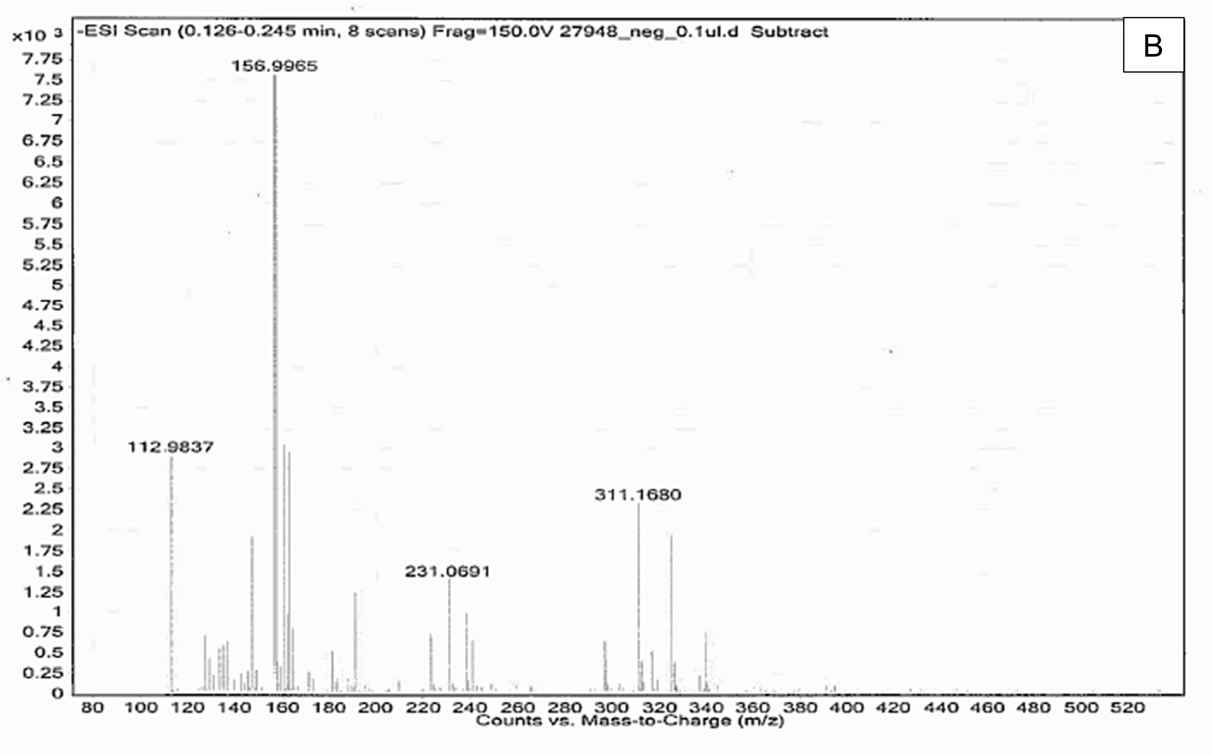

Figure S5. Electrospray ionization mass spectrometry (ESI-MS) spectra for [P_{66614}][BS], where A) is in positive mode and B) is in negative mode. Solvent used was DCM.

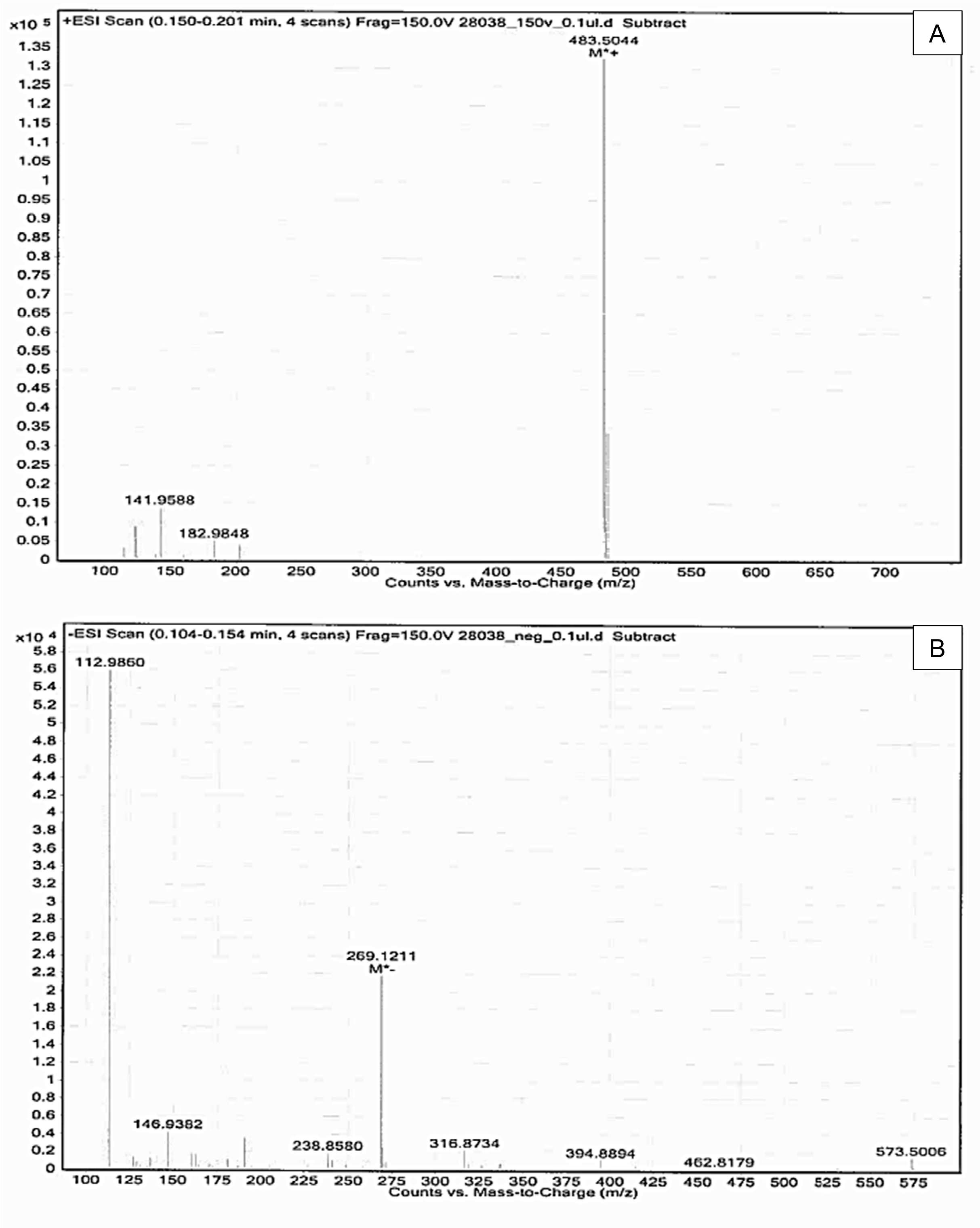

Figure S6. Electrospray ionization mass spectrometry (ESI-MS) spectra for [P_{66614}][OBS], where A) is in positive mode and B) is in negative mode. Solvent used was DCM.

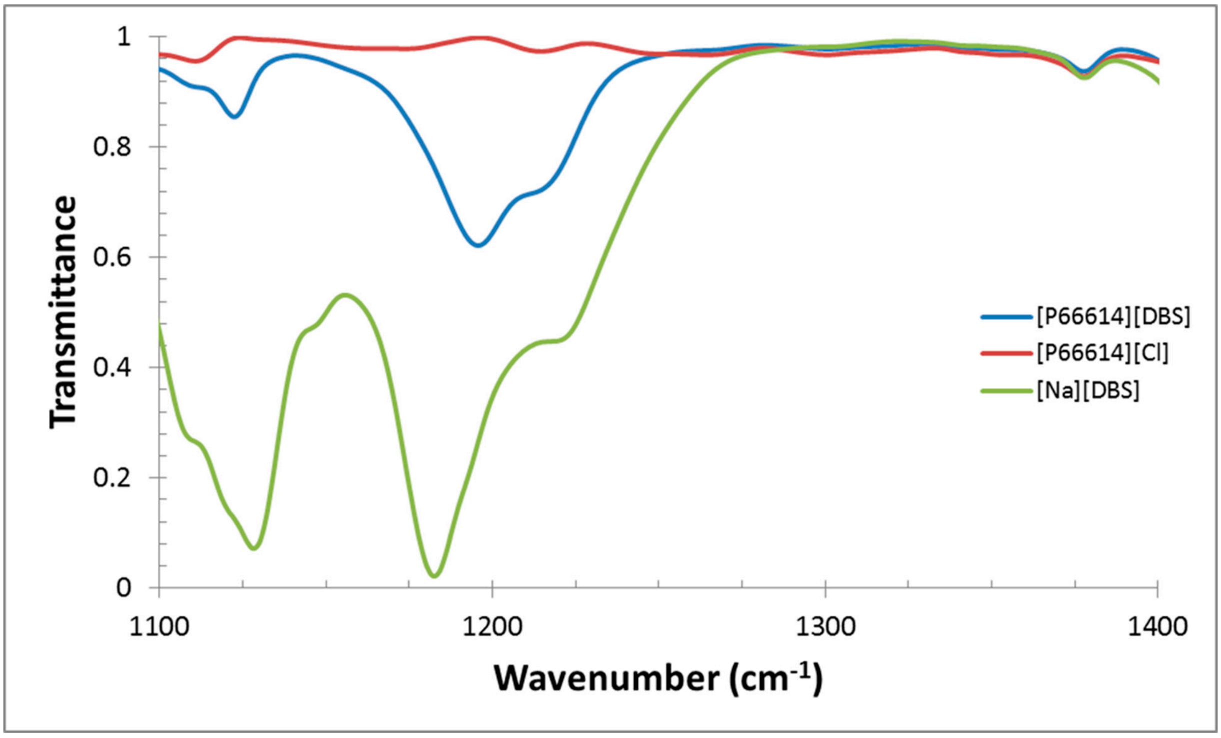

Figure S7. Fourier transform infrared (FT-IR) spectra for [P_{66614}][DBS]. S=O stretching in DBS ~1182 shifts to ~1195 in [P_{66614}][DBS].

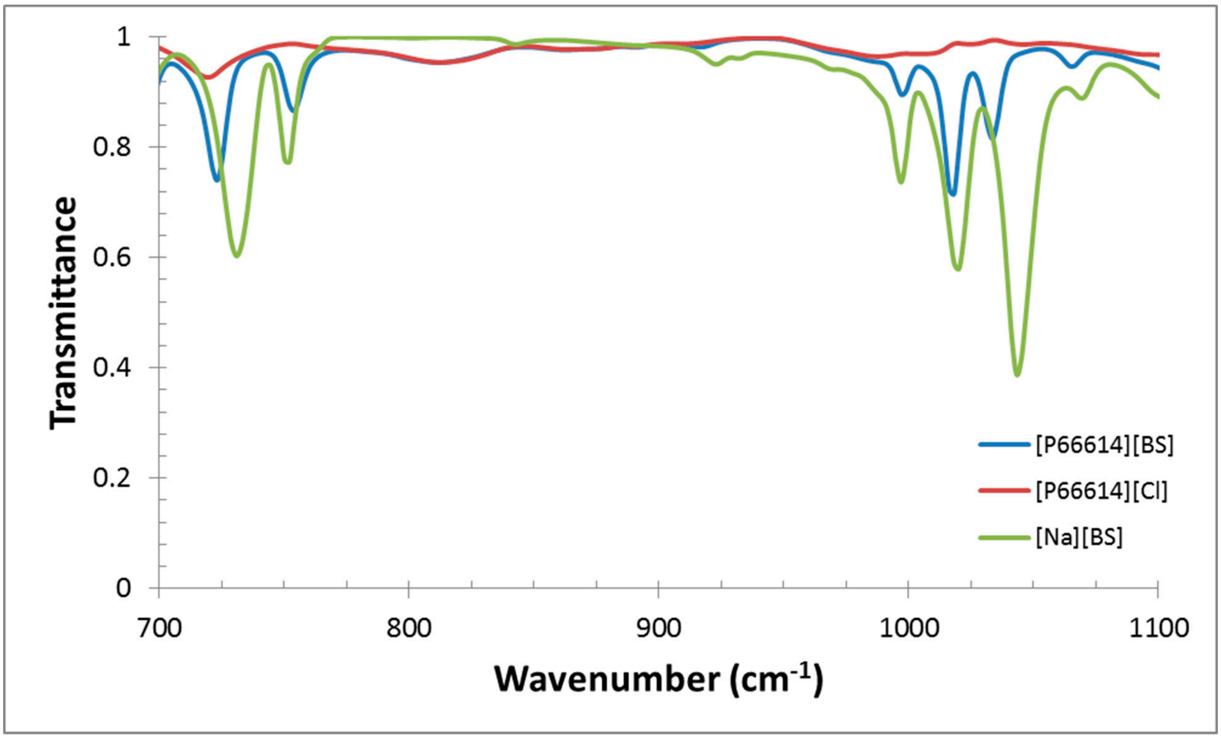

Figure S8. Fourier transform infrared (FT-IR) spectra for [P_{66614}][BS]. S-O stretching in BS ~732 shifts to ~725 and ~750 shifts to ~755 in [P_{66614}][BS].

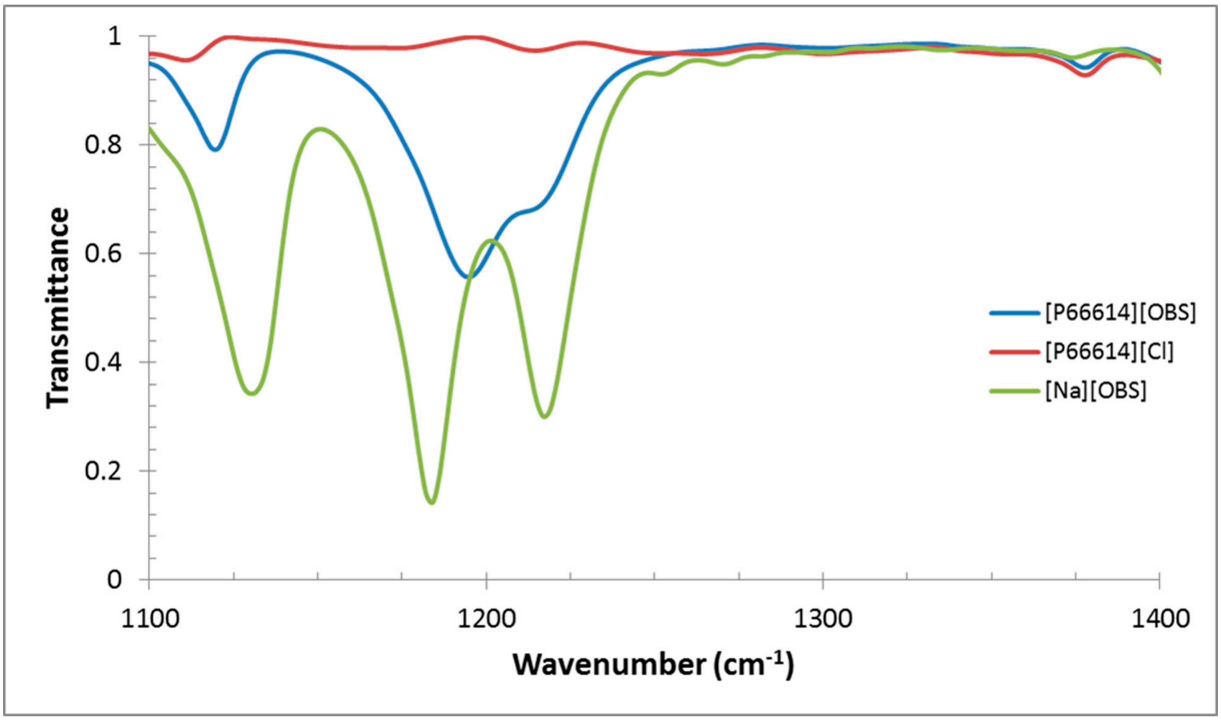

Figure S9. Fourier transform infrared (FT-IR) spectra for [P_{66614}][OBS]. S=O stretching in OBS ~1184 shifts to ~1195 in [P_{66614}][OBS].

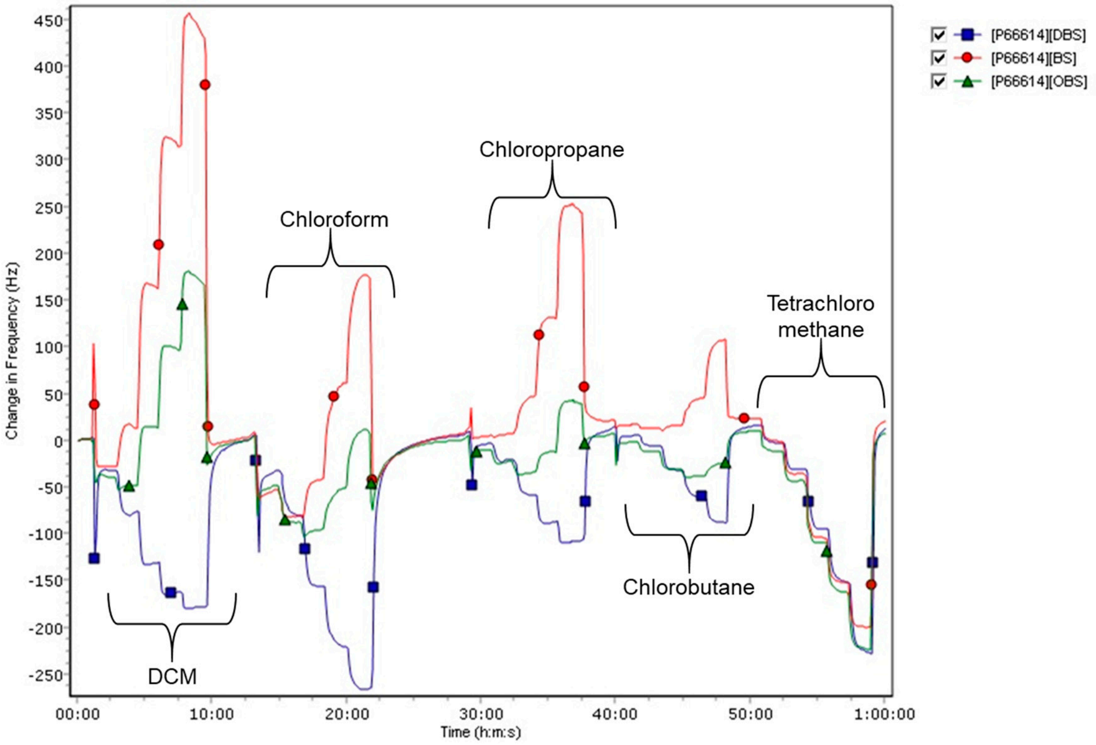

Figure S10. Sensorgram for first replicate measurement. Braces indicate the VOC being measured at multiple flow ratios in 3-minute increments.

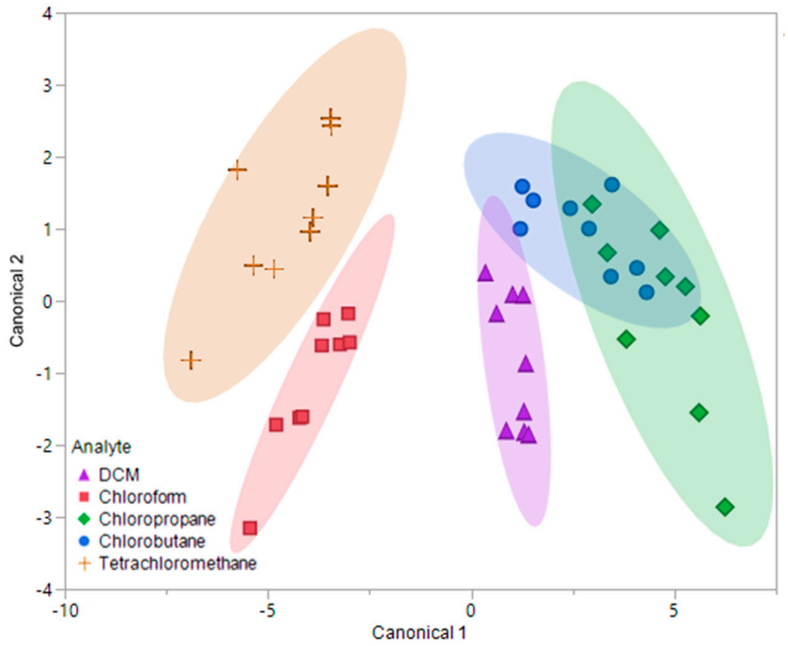

Figure S11. Canonical plot for discrimination of five chlorinated VOCs with respect to a five sensor VSA. Plot considers 45 total measurements consisting of three replicate measurements at three different flow ratios for each VOC (9 measurements per sample) using [P_{66614}][DBS]-PDMS.

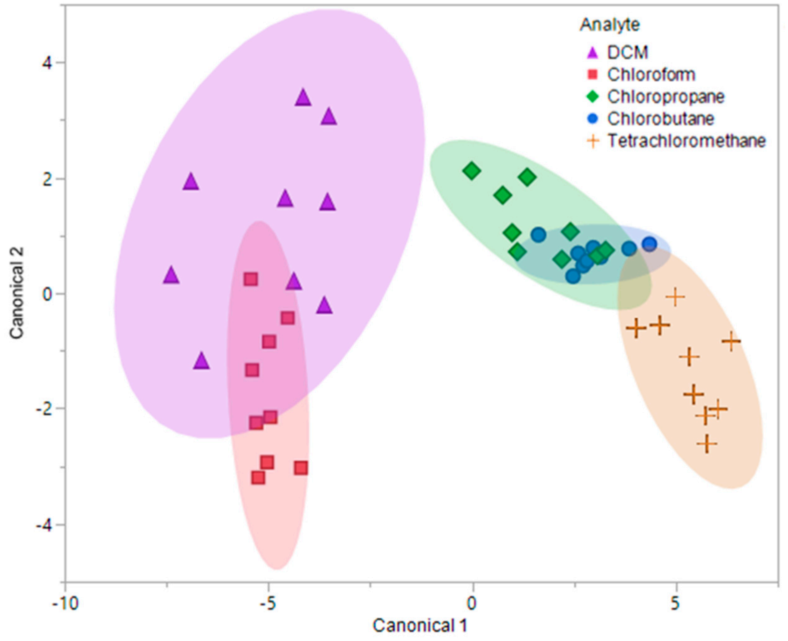

Figure S12. Canonical plot for discrimination of five chlorinated VOCs with respect to a four sensor VSA. Plot considers 45 total measurements consisting of three replicate measurements at three different flow ratios for each VOC (9 measurements per sample) using [P_{66614}][BS]-PDMS.

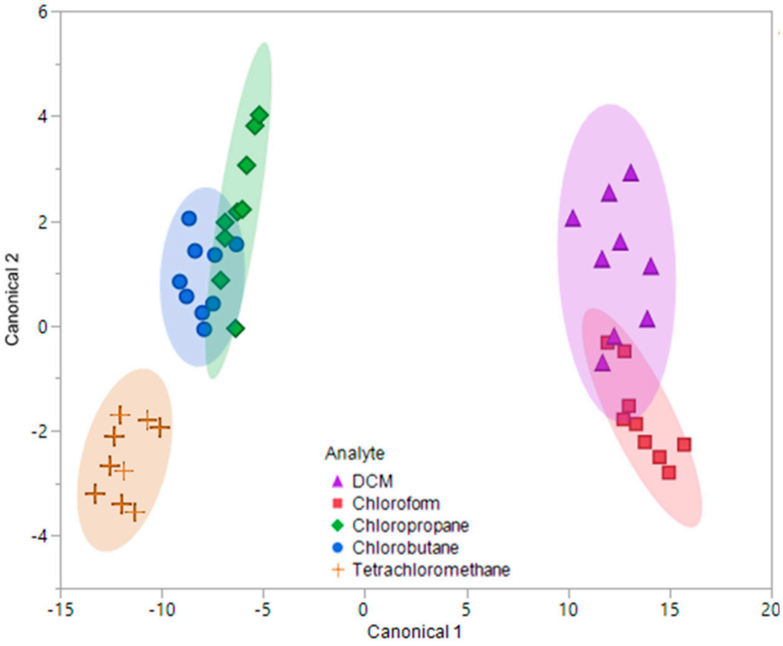

Figure S13. Canonical plot for discrimination of five chlorinated VOCs with respect to a six sensor VSA. Plot considers 45 total measurements consisting of three replicate measurements at three different flow ratios for each VOC (9 measurements per sample) using [P_{66614}][OBS]-PDMS.
